# Supplementary material for: Efficacy of Salmonella Bacteriophage S1 Delivered and Released by Alginate Beads in a Chicken Model of Infection
Source: Viruses. 2021 Sep 25;13(10):1932. doi: 10.3390/v13101932 (PMC8539449; doi:10.3390/v13101932)
Supplement: Supplementary file 1 [file viruses-13-01932-s001.zip › viruses-1372341-supplementary-done.pdf]

Supplementary Materials:

Table S1. Bacterial strains used in the present study.

Bacterium	Identification of bacterial
S. Enteritidis (SE PT13A)	Host strain
S. Typhi (ST-PH1)	ICTH
S. Pullorum (MDR-MC862-A)	ICTP
S. Gallinarum (MDR-MC862-B)	ICTP
S. Cholerasuis (SC-PH1)	ICTH
C. freundii (CF-PH1)	ICTH
E. cloacae (EC-PT1)	ICTH
E. coli (EC-PT1)	ICTH

ICTP: Isolated from clinical trials in poultry, ICTH: Isolated from clinical trials in humans

Table S2. Percentage of infectives bacteriophages post-exposure gastric conditions of poultry.

	pH 3 / 45 min	pH 5 / 15 min	pH 7 / 30 min	pH 8.5 / 20 min
**Anatomical portion simulated of *Gallus gallus domesticus***	**Proventriculus + gizzard**	**Duodenum**	**Jejunum e ileum**	**Cecum**
Non encapsulated bacteriophages*	38.23*	79.78*	96.37*	89.65*
Encapsulated bacteriophages**	14.95**	26.52**	46.30**	37.19**

* Percentage of infectivity of phage S1 non encapsulated; ** Percentage relative of phage S1 release from initial titer.
